# A clinical score to predict mortality in septic acute kidney injury patients requiring continuous renal replacement therapy: the HELENICC score

**DOI:** 10.1186/s12871-017-0312-8

**Published:** 2017-02-07

**Authors:** Rogério da Hora Passos, João Gabriel Rosa Ramos, Evandro Jose Bulhoes Mendonça, Eva Alves Miranda, Fábio Ricardo Dantas Dutra, Maria Fernanda R. Coelho, Andrea C. Pedroza, Luis Claudio L. Correia, Paulo Benigno Pena Batista, Etienne Macedo, Margarida M. D. Dutra

**Affiliations:** 1 Hospital Português, Salvador, Brazil; 2 Hospital São Rafael, Salvador, Brazil; 3 Faculdade de Medicina, Universidade de São Paulo, São Paulo, Brazil

**Keywords:** Septic acute kidney injury, Risk score, 7-day mortality, Hemodiafiltration

## Abstract

**Background:**

This study aimed to identify predictors of early (7-day) mortality in patients with septic acute kidney injury (AKI) who required continuous renal replacement therapy (CRRT).

**Methods:**

Prospective cohort of 186 septic AKI patients undergoing CRRT at a tertiary hospital, from October 2005 to November 2010.

**Results:**

After multivariate adjustment, five variables were associated to early mortality: norepinephrine utilization, liver failure, medical condition, lactate level, and pre-dialysis creatinine level. These variables were combined in a score, which demonstrated good discrimination, with a C-statistic of 0.82 (95% CI = 0.76–0.88), and good calibration (*χ* ^2^ = 4.3; *p* = 0.83). SAPS 3, APACHE II and SOFA scores demonstrated poor performance in this population.

**Conclusions:**

The HEpatic failure, LactatE, NorepInephrine, medical Condition, and Creatinine (HELENICC) score outperformed tested generic models. Future studies should further validate this score in different cohorts.

## Background

Sepsis and septic shock are the most common and severe causes of morbidity and mortality among critically ill patients [1]. Septic acute kidney injury (AKI) is one of the most life-threatening manifestations of sepsis [2, 3]. Septic AKI is associated with a high burden of illness, increased abnormalities in acute physiology and laboratory findings, and higher rates of non-renal organ failure and renal replacement therapy. Additionally, AKI has been shown to be independently associated with worse patient outcomes and increased health care costs [4].

Severity scores and risk stratification have been incorporated into the management of patients with AKI [5], because estimated probabilities of hospital mortality may provide important information for clinical decision-making [6], such as informing prognosis to patient and carers, discussions of goal-of-care, decisions about resource allocation and assessments of the quality of care that has been provided [6, 7]. However, it has been argued that general physiological scores may not have the same accuracy along the extended range of critical illnesses [8] and most general prognostic tools lack predictive accuracy or show great variability when applied to patients with AKI [9–12]. So, disease-specific scores have been developed in critically ill patients with AKI [13], but there are few data concerning the assessment of the severity of illness in septic AKI patients who undergo continuous renal replacement therapy (CRRT).

In this study, we aimed to identify predictors of early (7-day) mortality in septic critically ill patients undergoing CRRT. We also aimed to create a severity assessment tool capable of predicting the early mortality risk of septic critically ill patients at the start of CRRT and compare its performance with the performance of three generic scores (i.e., Simplified Acute Physiology Score [SAPS 3], Acute Physiology and Chronic Health Enquiry [APACHE II], and Sequential Organ Failure Assessment [SOFA]).

## Methods

This study was approved by the ethics committee at Hospital Português. Informed consent was waived because data were collected routinely and statistical analyses were performed anonymously.

### Study design and patient population

This prospective cohort study was conducted in three different intensive care units (cardiac, medical, and surgical units) at Hospital Portugues, a Brazilian tertiary hospital, from October 2005 to November 2010. All consecutive adult patients with septic AKI (as defined below) that would be submitted to continuous renal replacement therapy (CRRT), as indicated by the medical team, were included in the study. Patients expected to die in the following 24-h from the admission or patients that had limitation of medical therapies orders would not be candidates to CRRT and would not be included in the study. In the event of multiple admissions, only the first intensive care unit (ICU) admission was analyzed.

### Definitions

AKI was classified according to the Acute Kidney Injury Network (AKIN) criteria. Serum creatinine values were adjusted according to cumulative fluid balance from ICU admission, as described below. Sepsis was defined according to the International Sepsis Definitions Conference recommendations [14]. Septic AKI was defined as the simultaneous presence of both syndromes, sepsis and AKI, in the absence of other clear and established non-sepsis-related precipitants of AKI (i.e., urinary tract obstruction, radio contrast media, and other nephrotoxins). Comorbidity information was determined from the International Classification of Diseases (ninth revision) codes used upon admission [15]. Medical condition was defined as a medical (i.e., non-surgical) reason for admission in the ICU.

The primary endpoint was defined as early (7-day) mortality.

### Data collection

Patients’ demographics, primary diagnosis, and associated comorbidities were recorded at ICU entry. Demographic information included the patient’s age, sex, and admission dates. Clinical data comprised the source of infection, microbiological data, antibiotic use, fluid balance, need for mechanical ventilation, and use of vasopressor drugs (norepinephrine). Physiological data included the Glasgow Coma Scale score, vital signs, the arterial oxygen tension/fraction of inspired oxygen (PaO2/FiO2) ratio, and serum pH, sodium, potassium, bilirubin, hematocrit, and white blood cell count levels. Kidney function data included serum creatinine and urea levels, and urine output. Severity of illness was assessed on the day of the initiation of CRRT using the APACHE II, SAPS 3, and SOFA scores.

### Correction of serum creatinine for fluid balance

Admission weights were available in all patients. Daily fluid balance was determined from all intakes and outputs recorded. No correction was made for insensible losses. Cumulative fluid balance was computed by summing the daily fluid balances. Serum creatinine (sCr) values were adjusted according to the cumulative daily fluid balance using the formula [16, 17]:

$$ \begin{array}{l}\mathrm{adjusted}\ \mathrm{creatinine}=\mathrm{sCr}\times \mathrm{correction}\ \mathrm{factor}.\hfill \\ {}\mathrm{Correction}\ \mathrm{factor}=\left(\mathrm{hospital}\ \mathrm{admission}\ \mathrm{weight}\left(\mathrm{kg}\right)\times 0.6+\varSigma \left(\mathrm{daily}\ \mathrm{cumulative}\ \mathrm{fluid}\ \mathrm{balance}\left(\mathrm{L}\right)\right)\right)/\mathrm{hospital}\ \mathrm{admission}\ \mathrm{weight}\times 0.6.\hfill \end{array} $$

### CRRT procedure

CRRT was provided as continuous venous-venous hemodiafiltration (CVVHDF) and was started by consulting nephrologists based on standard clinical guidelines, including AKI with hemodynamic instability, ongoing hypercatabolism, hyperkalemia, severe acidosis, volume overload, respiratory distress, multiorgan failure or some combination of these factors. Saline flushes were used to maintain filter patency, instead of heparin. Patients were routinely treated by CVVHDF with bicarbonate buffered solution. The procedure was performed using the Gambro PRISMA continuous renal replacement therapy (CRRT) machine. In all patients, a M100 hemofilter was used and was routinely changed after 72 h. The ultrafiltrate flow rate was set accordingly daily fluid balance gains. The prescribed dialysis dose was around 20 a 30 ml/kg/h, as per institutional protocol.

### Statistical analysis

Statistical analysis was performed using IBM SPSS® Statistics version 18.0. Continuous variables are expressed as the mean (SD) or median and interquartile range, and were analyzed using the unpaired *t*-test or the Wilcoxon rank-sum test, as appropriate. Categorical variables are expressed as the absolute (n) and relative (%) frequency, and were analyzed using the chi-squared test or Fisher’s exact test, as appropriate. Data are presented with 95% confidence intervals (CIs) and *p* < 0.05 was considered to be statistically significant for all comparisons.

A logistic regression model was constructed to evaluate variables independently associated to 7-day mortality and for the construction of a prediction score. Initially, univariate analysis was performed to identify variables associated with 7-day mortality. Variables associated to early mortality with a *p* value <0.10 in univariate analysis were selected for logistic regression analysis, which was performed by a stepwise procedure with backward elimination using Wald statistic. The independent predictors of mortality at a 5% significance level were used to build a risk score. The risk score was built by assigning points to each variable, corresponding to their odds ratio, multiplied by 2, and rounded to the nearest integer. To describe the predictive value of the score, C-statistics were used for discrimination and the Hosmer-Lemeshow test was used for calibration. We also analyzed the area under the receiver operating characteristic curve to evaluate the performance of the score, as compared to the other generic predictive scores.

## Results

### Description of the cohort

From October, 2005 to November, 2010, 186 septic AKI patients undergoing CVVHDF were enrolled in the study. There were 84 (45%) patients in the surgical ICU, 61 (33%) patients in the medical ICU and 41 (22%) patients in the cardiac ICU. Mean age ± SD was 68.7 ± 14.6 years and 104 (56%) patients were male. The most common source of infection was pulmonary infection (56% of patients), followed by bloodstream infection in 16% of patients. Hospital acquired sepsis accounted for 74% the cases and community acquired sepsis for 26%. On the day of CRRT initiation, mean ± SD for SAPS 3, APACHE II, and SOFA scores were 74.1 ± 9.9, 28.5 ± 8.3, and 12.7 ± 2.4, respectively. Mortality at 7 days was 45% (84 patients).

### Characteristics associated to 7-day mortality

In univariate analysis of the clinical and laboratory characteristics, nine variables were associated with 7-day mortality at the 10% significance level: liver failure, heart failure, medical condition, norepinephrine utilization, pre-dialysis lactate level, sodium level, potassium level, creatinine level, and platelet count (Table 1).

**Table 1:** Association of clinical characteristics and 7-day mortality in the cohort

	Non Survivors	Survivors	*p* value
	(*n* = 84)	(*n* = 102)	
Age (years), mean ± SD	67 ± 16	70 ± 13	0.14
Male sex, n (%)	42 (50%)	62 (61%)	0.14
Medical condition, n (%)	57 (68%)	50 (49%)	0.01
Primary site of infection, n (%)			
Pulmonary	49 (58%)	55 (54%)	0.55
Urinary	6 (7.1%)	9 (8.8%)	0.68
Abdominal	8 (9.5%)	12 (12%)	0.62
BSI ^a^	14 (17%)	15 (15%)	0.71
CRBSI ^b^	5 (6.0%)	11 (1%)	0.24
SSTIs ^c^	2 (2.4%)	0	0.20
Comorbidities, n (%)			
Heart failure	28 (33%)	50 (49%)	0.03
Coronary artery disease	25 (30%)	42 (41%)	0.11
Pulmonary disease	8 (9.5%)	10 (9.8%)	1.0
Liver failure	55 (66%)	32 (31%)	<0.001
Cerebral vascular disease	7 (8.3%)	8 (7.8%)	0.90
Diabetes	23 (27%)	37 (36%)	0.20
Norepinephrine use	78 (93%)	80 (78%)	0.006
Admission creatinine (mg/dl), median (IQR)	1.0 (0.8–1.6)	1.2 (0.9–2.2)	0.14
Laboratory values before RRT ^d^, median (IQR)			
Urea (mg/dl)	142 (86–198)	137 (78–206)	0,57
Chloride (mmol/l)	109 (104–112)	107 (104–110)	0,41
Creatinine (mg/dl)	2.2 (1.5–3.3)	2.7 (2.0–3.8)	0.005
Potassium (mmol/l)	4.7 (3.8–5.4)	4.3 (3.8–5.0)	0.05
Sodium (mmol/l)	133 (127–137)	135 (132–139)	0.009
Lactate (mmol/l)	3.0 (1.8–5.1)	1.8 (1.4–2.7)	<0.001
Hemoglobin (g/dl)	8.9 (8.0–9.9)	9.1 (8.2–10)	0.21
White cell count (10^3^ mm^3^/l)	17 (6.6–24)	16 (11–22)	0.64
Platelets count (10^3^ mm^3^/l)	94 (48–176)	155 (79–265)	0.001
24 h urinary output (ml/kg/h)	320 (150–600)	300 (100–600)	0.57
Fluid Balance (ml)	7782 (4175–11,389)	6891 (4090–9692)	0.007

^a^ *BSI* bloodstream infections

^b^ *CRBSI* cateter-related bloodstream infections

^c^ *SSTIs* skin and soft tissue infections

^d^ *RRT* renal replacement therapy

After multivariate analysis, five variables remained significantly associated to mortality (Table 2): norepinephrine utilization, liver failure, medical condition, lactate and pre-dialysis creatinine. This final model had a significant C-statistic of 0.82 (95% CI = 0.76–0.88) and good calibration (*χ* ^2^ = 4.3; *p* = 0.83).

**Table 2:** Multivariate analysis of characteristics associated to 7-day mortality

Variable	B	OR (95% CI)	*p* value
Norepinephrine use	1.116	3.1 (1.1–8.7)	0.038
Lactate (mmol/l)	0.480	1.6 (1.3–2.1)	<0.001
Pre-dialysis creatinine (mg/dl)	−0.337	0.71 (0.55–0.93)	0.013
Liver failure	0.978	2.7 (1.3–5.4)	0.007
Medical condition	0.988	2.7 (1.3–5.6)	0.008

### Score development and performance evaluation

Based on the magnitude of regression coefficients in the multivariate analysis, we created the HEpatic failure, LactatE, NorepInephrine, medical Condition, and Creatinine (HELENICC) score. Score points were attributed proportionally to the odds ratio for each variable, thereby generating a scoring system. Medical condition, liver failure, and norepinephrine utilization each added 6 points, and 3 points was added for each unit of lactate. Two points were subtracted for each unit of creatinine. The scoring system was derived as follows: (medical condition × 6) + (liver failure × 6) + (norepinephrine × 6) + (lactate × 3) − (creatinine × 2).

**Fig. 1:**
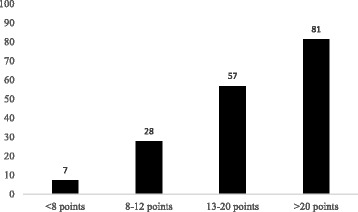
Seven-day mortality (%) in each of HELENICC score’s quartiles (chi-square for trend, *p* < 0.001)

**Fig. 2:**
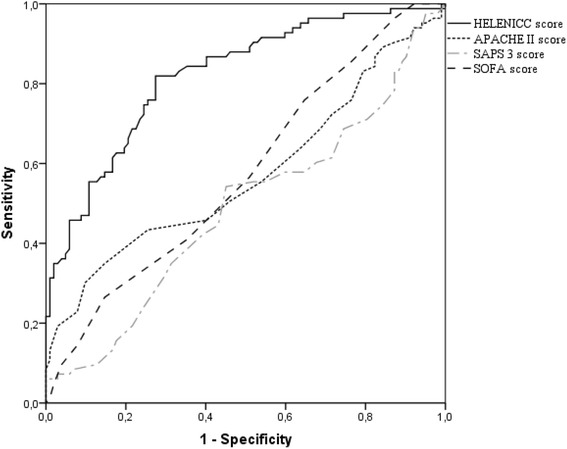
Area under the ROC curve for the HELENICC score (0.82; 95% CI = 0.76–0.88); APACHE II score (0.57; 95% CI = 0.48–0.66); SAPS 3 score (0.48; 95% CI = 0.40–0.57) and SOFA score (0.58; 95% CI = 0.49–0.66)

The HELENICC score was linearly associated to mortality (chi-square for trend, *p* <0.001), as seen in Fig. 1, with each increasing score quartile being associated to mortality with an OR (95% CI) of 3.15 (2.29–4.45). The score also demonstrated good discrimination, with an area under the ROC curve of 0.82 (95% CI = 0.76–0.88; *p* < 0.001), (Fig. 2). This performance was better than that of the generic scores, as the area under the receiver operating characteristic (ROC) curve was not significant for SAPS 3 (area under the curve [AUC] = 0.48; 95% CI = 0.40–0.57; *p* = 0.08), APACHE II (AUC = 0.57; 95% CI = 0.48–0.66; *p* = 0.10), or SOFA (AUC = 0.58; 95% CI = 0.49–0.66; *p* = 0.58) (Fig. 2).

## Discussion

In this prospective cohort of 186 critically ill patients with septic AKI undergoing CRRT, norepinephrine use, lactate, liver failure, medical condition and pre-dialysis creatinine were independently associated to 7-day mortality. Those characteristics were used to build a scoring system to predict early mortality (the HELENICC score), which demonstrated good discrimination and calibration and outperformed generic scoring systems in this population.

The HELENICC score modeling identified five independent variables (norepinephrine utilization, medical condition, liver failure, pre-dialysis lactate, and creatinine) that were associated with mortality, allowing the assessment of septic AKI requiring CRRT. The HELENICC score demonstrated good calibration, as assessed by the Hosmer-Lemeshow statistic, and was linearly associated to mortality with an odds ratio for mortality of approximately 3 for each increased quartile. Moreover, the HELENICC score demonstrated good discrimination, with an area under the ROC curve of 0.82, which was better than the other generic scores tested.

Even more, this score reflects the same stage of disease progression, and it was performed on the day of CRRT initiation, as carried out in previous studies [9, 11]. Another study on a large and diverse AKI population spanning three hospitals in a health system and including all patients regardless of their ICU, surgical, or medical status, showed that a small group of easily measured clinical factors had good ability to predict mortality and the need for dialysis in severe AKI [18]. As with the HELENIC score, this previous study identified strong predictors (e.g., intensive care unit location, medical service, liver disease, creatinine level and value variation, and greater number of pressor medications) of the combined endpoint of dialysis or death within 14 days [18].

A potential strength of the new score is its simplicity. This score is based on easily available variables, all with known effects on AKI mortality, and allows risk stratification of patients into different severity levels with progressive rates of mortality. There are important theoretical advantages to disease-specific predictive models [9]. The application of generic severity scores in specific cohorts, such as in patients with septic AKI, is challenging because of relatively poor discrimination and suboptimal calibration [19]. Almost all generic severity scores include “points” for AKI; in an AKI cohort, these points are equally assigned to all patients, limiting the value of the information [20, 21]. In fact, all generic models tested (i.e., APACHE II, SAPS, 3 and SOFA) in the present study showed similar performance levels, but significantly underestimated the mortality rate. Uchino et al. [5] showed the same results as in our study after testing two general scoring systems (SAPS II and SOFA) in a cohort of 1742 intensive care unit patients from multiple countries with AKI. These patients were either treated with renal replacement therapy or fulfilled the predefined criteria. In their study, none of the tested scoring systems demonstrated a high level of discrimination or calibration for predicting mortality in patients with AKI [5]. Other studies demonstrated variable results. Douma et al. [10] showed that the predictive abilities of several mortality prediction models varied widely when applied to AKI patients undergoing dialysis. Costa e Silva et al. [11] reported that the Simplified Acute Physiology Score (SAPS) 3 was the most accurate scoring system among third generation models for predicting hospital mortality in AKI. In a cohort of critically ill dialytic AKI patients, Maccariello et al. [12] showed that the SAPS 3 accurately predicted hospital mortality at the start of renal replacement therapy.

In our population, we decided to study early mortality (7-day mortality) from the beginning of CRRT, because our patients were severely ill, with high APACHE II scores and SAPS 3, as well as high organ dysfunctions as measured with the SOFA score. This severity was associated with high early mortality and such risk assessment is important to help inform the clinicians, patients and surrogates involved in the patient care. Our mortality results are consistent with the results of a large multicenter observational cohort [22]. In this cohort, Bagshaw et al. [22] described the characteristics and investigated the clinical outcomes of septic and non-septic AKI critically ill patients. Septic AKI was associated with a greater burden of illness, as shown by the severity-of-illness scores, concomitant non-renal organ dysfunction, need for mechanical ventilation, and proportion of patients who required vasoactive therapy. In their study, septic AKI was associated with a higher crude in-hospital case-fatality rate than non-septic AKI (70.2% versus 51.8%; relative risk, 1.35; 95% CI = 1.3–1.5; *p* < 0.001) [22].

In our study, higher creatinine levels at the start of CRRT showed a protective effect. Cerdá et al. [23] obtained the same results as in our study in a cohort of 134 critically ill patients with AKI who required CRRT. Multivariate logistic regression analysis detected a relationship between a higher serum creatinine level at CRRT initiation and improved survival rate (odds ratio, 1.48: 95% CI = 1.034–1.999) [23]. To avoid the probable effect of fluid balance on serum creatinine levels, we corrected creatinine levels using the formula suggested by Macedo et al. [17]. In a cohort of 253 patients recruited from a prospective observational study of critically ill patients with AKI, they showed that dilution of creatinine by fluid accumulation may lead to underestimation of the severity of AKI and increase the time required to identify a 50% relative increase in creatinine levels.

Our study has several limitations. Recently, the third international consensus for sepsis and septic shock has been proposed [24] with new definitions for those syndromes. However, even though our data was collected before the release of this consensus, our inclusion criteria of septic patients as patients with infection and a new infection-related organ dysfunction (i.e., acute kidney injury) is in agreement with those new definitions. Other limitation is that there were no data for assessing compliance to early goal directed therapy, or any other measures of processes of care, in this group of patients before or during CRRT, which can biases our results. For example, Plataki et al. [25] reported that, in a cohort of 390 patients with septic shock admitted to a medical ICU, patients and health care delivery risk factors appeared to be important factors for the development of AKI. Even more, our database did not include any data on the exact indications, and the rate of ultrafiltration and timing for CVVHDF.

Moreover, although the same medical team cared for all patients in this study, there was inevitable day-to-day variation in the practitioners who were in charge of the patients. These changes in the process of care might have induced some variability in the quality of care, which could have caused a degree of bias in the results. For example, there were no objective criteria for initiation of CRRT and there has been wide controversies about the benefits of early versus delayed initiation of renal replacement therapy in critically ill patients [26], especially after the publication of two large randomized controlled trials with divergent results [27, 28]. So, it is possible that the HELENICC score may perform differently in patients submitted to early or delayed initiation of renal replacement therapy. However, although we lack specific data, based on the routine indications for CRRT in our service, it is reasonable to infer that most patients in this study would have been classified as delayed initiation of renal replacement therapy in the ELAIN trial [27], although many of them might have been classified as early initiation in the AIKIKI trial [28].

Furthermore, because we aimed to analyze early (7-day mortality), we did not present data on long term mortality or other relevant functional outcomes, so there is no information on the performance of the score for those outcomes. Additionally, as with any probabilistic score, the use of such probabilities to assess prognosis for individual patients demands caution, especially when such predictions may have an impact on the provision of care to the individual patients, because of the high level of uncertainty implicated on such analyzes [6]. Finally, although this score has been prospectively developed at our institution in three different ICUs, this was a single-center study with limited generalizability and this model or any such predictive model should be independently validated in other populations and at any other institutions that propose its use.

## Conclusion

In summary, in this study we developed a scoring system that accurately predicts 7-day mortality. This model outperformed generic models in this population. Future studies should further validate this score in different cohorts.

## Abbreviations

AKI: Acute kidney injury
ICU: Intensive care unit
RRT: Renal replacement therapy
CRRT: Continuous renal replacement therapy
CVVHDF: Continuous venovenous hemodiafiltration
SAPS3: Simplified Acute Physiology Score
APACHE II: Acute Physiology and Chronic Health Enquiry
SOFA: Sequential Organ Failure Assessment
HELENICC: The HEpatic failure, LactatE, NorepInephrine, medical Condition, and Creatinine score
